# Evaluating the safety and feasibility of prophylactic third-party NK cell administration in high-risk AML patients post-HSCT

**DOI:** 10.1186/s12885-025-15362-8

**Published:** 2025-11-22

**Authors:** Maryam Barkhordar, Shima Tavoosi, Shirin Tavakoli, Maryam Samareh Salavatipour, Tanaz Bahri, Bahram Chahardouli, Amir Hossein Baghsheikhi, Mohammad Vaezi, Davoud Babakhani, Soroush Rad, Seied Asadollah Mousavi, Ghasem Janbabai, Hossein Salehi-Shadkami, Mohammad Ahmadvand

**Affiliations:** 1 Hematologic Malignancy Research Center, Research Institute for Oncology, Hematology and Cell Therapy, Tehran University of Medical Sciences, Tehran, Iran; 2 Department of Cell and Molecular Biology and Microbiology, Faculty of Biological Science and Technology, University of Isfahan, Isfahan, Iran; 3 Department of Applied Cell Sciences, School of Advanced Technologies in Medicine, Tehran University of Medical Sciences, Tehran, Iran; 4 Cell Therapy and Hematopoietic Stem Cell Transplantation Research Center, Research Institute for Oncology, Hematology and Cell Therapy, Tehran University of Medical Sciences, Tehran, Iran; 5 Department of Biology, Science and Research Branch, Islamic Azad University, Tehran, Iran; 6 Tehran university of medical sciences, Tehran, Iran

**Keywords:** Natural killer cells; acute myeloid leukemia, Immunotherapy, Graft-versus-host disease

## Abstract

**Background:**

Relapse is a major cause of treatment failure in high-risk acute myeloid leukemia (AML) after hematopoietic stem cell transplantation (HSCT). Natural killer (NK) cell immunotherapy may enhance graft-versus-leukemia (GvL) effects without increasing graft-versus-host disease (GvHD). This study assessed the safety and feasibility of early post-HSCT prophylactic infusions of third-party NK cells in high-risk AML.

**Methods:**

In a single-arm, non-randomized trial, 11 high-risk AML patients received two doses of ex vivo expanded third-party NK cells (5 × 10⁶ cells/kg) on days 6 and 12 post-HSCT. Endpoints included safety (CTCAE v5.0), relapse incidence, overall survival (OS), and disease-free survival (DFS). NK cell products were assessed for purity (≥ 80% CD56⁺CD3⁻), cytotoxicity (K562 assay), and expansion.

**Results:**

NK cell infusion was well tolerated, with no grade 3 or higher infusion-related toxicities. Acute GvHD (Grade 1–2) occurred in 36.4% (4/11); chronic GvHD in 27.3% (3/11). CMV reactivation occurred in 45.5% (5/11) and was managed preemptively. At a median 256-day follow-up (54–514), Relapse occurred in 27.3% (3/11; median: 111 days). Survival was significantly better in patients in CR1/CR2 at HSCT (83.3%) compared to those not in remission (20%; *p* = 0.02).

**Conclusion:**

Early prophylactic NK cell infusions post-HSCT are safe and feasible. Although relapse incidence remains substantial, outcomes appear improved versus historical data. Future randomized trials must confirm clinical benefits and refine timing/dosing strategies.

**Supplementary Information:**

The online version contains supplementary material available at 10.1186/s12885-025-15362-8.

## Background

Acute myeloid leukemia (AML) is an aggressive hematologic malignancy driven by the uncontrolled proliferation of immature myeloid cells [1]. In 2021, the reported incidence of AML in adults was 4.2 per 100,000 [2]. While frontline therapies, including chemotherapy, radiation, and targeted agents, induce remission in many patients, relapse remains a critical barrier to long-term survival, particularly in high-risk cohorts [3].

Allogeneic hematopoietic stem cell transplantation (allo-HSCT) remains the cornerstone of curative intent, leveraging graft-versus-leukemia (GvL) effects to eliminate residual disease [4, 5]. However, even with allo-HSCT, relapse persists as the leading cause of mortality, affecting 30–60% of recipients [6].

To mitigate relapse, strategies such as NK cell-based immunotherapy are increasingly explored to augment GvL effects without exacerbating graft-versus-host disease (GvHD) [7]. Unlike T cells, which mediate GvHD, NK cells exert early antitumor activity post-HSCT, potentially bridging the immune gap before full donor lymphocyte reconstitution [8]. Natural killer (NK) cells, constituting 5–15% of circulating lymphocytes, are innate immune effectors capable of lysing malignant and stressed cells without prior sensitization [9, 10]. Their activity is governed by a balance of activating (e.g., *NKG2D*, *NCRs*) and inhibitory receptors (e.g., *KIR*, *NKG2A*), which recognize stress ligands and MHC class I molecules, respectively [11]. This unique biology enables NK cells to target leukemia blasts with downregulated MHC-I, a common immune evasion mechanism in AML [12].

While donor-derived NK cells reduce relapse in early-phase trials [13]. logistical barriers, including donor availability and prolonged manufacturing, limit scalability [14]. Third-party NK cells, cryopreserved and readily available, present an ‘off-the-shelf’ solution to these challenges. However, their efficacy in high-risk AML post-HSCT remains underexplored. This pilot study evaluates the feasibility and safety of prophylactic third-party NK cell infusions in this setting, addressing a critical unmet need.

## Materials and methods

### Study design and patients

This single-arm, open-label pilot trial (IRCT20140818018842N35, Registration date: 2023-07-13) was conducted at the Hematology-Oncology-Stem Cell Transplantation Research Center (HORCSCT) of Tehran University of Medical Sciences. Eligible patients were adults (≥ 18 years) with high-risk AML per the 2022 European LeukemiaNet (ELN) criteria [15], characterized by adverse-risk cytogenetics (e.g., complex or monosomal karyotype), FLT3-ITD or TP53 mutations, persistent pre-HSCT measurable residual disease (MRD ≥ 0.1%), primary induction failure, or early relapse (< 12 months). Exclusion criteria comprised active infections, prior NK cell therapy, or severe organ dysfunction (e.g., left ventricular ejection fraction < 40% or creatinine clearance < 30 mL/min).

### Conditioning regimen before HSCT

Patients received a myeloablative conditioning regimen of intravenous fludarabine (30 mg/m²/day, days − 6 to −2) and busulfan (3.2 mg/kg/day, days − 5 to −2). GvHD prophylaxis comprised cyclosporine (2.5 mg/kg BID, target trough 150–250 ng/mL) and methotrexate (15 mg/m² on day + 1, 10 mg/m² on days + 3, +6, + 11), as per institutional protocols [16, 17].

### Third-party NK cell manufacturing and administration

The third-party NK cell products were derived from different non-related healthy volunteers who provided informed consent. To ensure the safety of the cellular products, all donors were screened and excluded if they tested positive for HIV, HBV, HCV, or had a positive blood culture, in accordance with standard blood bank and Good Manufacturing Practice (GMP) guidelines. donors selected for the “mismatch” at HLA loci to drive favorable NK cell–mediated effects. As Parham et al. reported previously, only a minority of HLA-matched transplants are KIR matched [18].

Third-party healthy donors’ peripheral blood mononuclear cells (PBMCs) were extracted using Ficoll Paque Premium (GE Healthcare, USA) and density gradient centrifugation at 300 g for 20 min. The final mixture was separated into three layers. The middle layer was collected using a pipette, transferred to a different tube, and washed twice with phosphate buffer saline (PBS) preheated to 25 °C. The harvested cell pellets were re-suspended in 1 mL of RPMI-1640 medium (Gibco, USA), followed by an assessment of cell viability through visual inspection and enumeration using 4% trypan blue solution (Sigma, Germany).

**Fig. 1:**
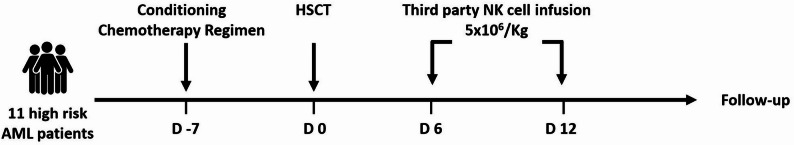
Study design timeline for third-party NK-Cell administration post-transplant. 11 high risk AML patients received 2 doses of third-party NK cell infusion at day 6 and day 12 post HSCT

Highly purified NK cells were isolated following the manufacturer’s protocol (Miltenyi Biotec, Germany), with non-NK cell populations removed via magnetic-activated cell sorting (MACS). For the expansion of NK cells, isolated cells were seeded at a density of 5 × 10⁵ cells/mL and co-cultured with irradiated (100 Gy) K562 cells genetically engineered to support NK cell proliferation (5 × 10⁶ cells/mL) in T-25 culture flasks. Cultures were maintained in RPMI-1640 medium (Gibco, USA) supplemented with 1000 IU/mL interleukin-2 (IL-2; Miltenyi Biotec, USA), 10 ng/mL interleukin-15 (IL-15), and 5% human serum (Sigma-Aldrich). On day 5, proliferating NK cells were transferred to larger culture flasks containing fresh RPMI-1640 medium supplemented with the same concentrations of IL-2, IL-15, and human serum. The culture medium was replenished every 2–3 days with fresh cytokine-supplemented medium for a total of 21 days. All NK cell expansion procedures were performed under conditions compliant with Good Manufacturing Practice (GMP) standards. Cryopreserved NK cells were thawed and infused intravenously (5 × 10⁶ cells/kg) on days + 6 and + 12 post-HSCT to coincide with early immune reconstitution (Fig. 1).

### Characterization and functional assessment of NK cell products

To determine the purity of natural killer (NK) cells isolated from peripheral blood (PB-NK), the relative proportions of CD3-negative and CD56-positive populations were quantitatively analyzed using flow cytometry (FACSCalibur; Becton Dickinson, USA). For this purpose, approximately 1 × 10⁵ cells were collected, washed with phosphate-buffered saline (PBS), and incubated for 20 min at ambient temperature in the dark with fluorochrome-conjugated monoclonal antibodies specific for CD56 (APC-labeled) and CD3 (FITC-labeled) (BioLegend, USA). After staining, cells were subjected to additional PBS washes to remove unbound antibodies. The stained cell populations were then analyzed on a flow cytometer, and data were processed using FlowJo software to quantify the expression profiles of CD3 and CD56 markers (Figure S1). The viability of the expanded cells was determined using the trypan blue exclusion assay. The cytotoxicity of purified NK cells was evaluated against the K-562 cell line, as in our previous study (Figure S2).

### Endpoints and statistical analysis

The primary endpoint was safety, graded per CTCAE v5.0. Secondary endpoints included relapse incidence, overall survival (OS), and disease-free survival (DFS). Continuous variables were summarized as median (range), and categorical variables as frequencies (%). OS and DFS were analyzed using Kaplan-Meier curves, with comparisons via the log-rank test. Relapse incidence was calculated via cumulative incidence analysis, accounting for competing risks (e.g., non-relapse mortality). All analyses were performed using SPSS v28 (IBM), with significance set at *p* < 0.05.

## Results

### Patients, disease characteristics, and treatment schedule

As depicted in Table 1, the study included 11 high-risk AML patients (median age: 45 years, range: 17–54; 72.7% male). All patients harbored adverse-risk features per ELN 2022 criteria [15]. Cytogenetic abnormalities predictive of poor prognosis, Monosomy 7, inv (3) (q21q26), and t (6;9) (p23; q34), were identified in 27.3% (*n* = 3/11) of patients.

**Table 1:** Baseline clinical and demographic characteristics of High-Risk AML patients

Patient	Age	Sex	Cytogenetic Abnormality	NPM1	FLT3_ITD	Remission Status at HSCT	MRD Status at HSCT	Donor Type	CD3 (×10⁶/kg)	CD34 (×10⁶/kg)
P-1	50	male	N/A	N/A	N/A	CR2	0.6%	Full-match	280.00	7.00
P-2	35	male	inv (3) (q21q26)	N/A	Wild Type	PIF and CR1	Negative	Full-match	164.00	7.50
P-3	17	male	t (6;9) (p23; q34)	Mutant	Mutant	CR2	0.8%	haploidentical	218.00	8.28
P-4	45	male	NL karyotype	Wild Type	Mutant	PIF and CR2	Negative	haploidentical	262.00	9.57
P-5	45	male	NL karyotype	Wild Type	Wild Type	Not in Remission	9%	haploidentical	243.00	8.54
P-6	44	female	NL karyotype	N/A	N/A	Not in Remission	6%	Full-match	221.00	7.82
P-7	51	male	NL karyotype	Wild Type	Wild Type	CR1	0.9%	haploidentical	325.00	10.00
P-8	27	female	NL karyotype	N/A	N/A	CR1	0.2%	Full-match	172.00	8.00
P-9	54	male	N/A	N/A	N/A	CR1	0.3%	Full-match	280.00	6.00
P-10	43	male	Monosomy 7	N/A	N/A	Not in Remission	5.24%	Full-match	161.50	7.00
P-11	52	female	NL karyotype	Wild Type	Wild Type	Not in Remission	3%	Full-match	275.00	5.50

*HSCT *Hematopoietic stem cell transplantation, *CR* Complete response, *PIF* Primary induction failure, *NL *Normal, *N/A* Data not available or not tested, *MRD* Measurable Residual Disease

Recipient ages ranged from 17 to 54 years (median: 45 years), with 8 males (72.7%) and 3 females (27.3%). Donors included siblings (*n* = 8), offspring (*n* = 2), and second-degree relatives (*n* = 1), with donor ages spanning 10–58 years. Donor types were evenly distributed between full-matched (*n* = 6) and haploidentical (*n* = 5) transplants.

At HSCT, 63.6% of patients (*n* = 7/11) were in complete remission (CR1/CR2), while 36.4% (*n* = 4/11) were not in remission. Pre-transplant measurable residual disease (MRD) was undetected only in 18.2% of patients (*n* = 2/11).

### NK-cell functionality and safety

The third-party NK cells used in this study demonstrated high purity and functionality, as evidenced by their potent cytotoxicity against the K562 cell line. These NK cells were shown to effectively eliminate target cells, highlighting their potential as a therapeutic agent. In terms of safety, the infusion of third-party NK cells in high-risk AML patients was associated with a low-grade and manageable adverse event profile. Adverse events were graded according to the National Cancer Institute Common Terminology Criteria for Adverse Events (NCI CTCAE). The most common adverse events included sinus tachycardia (grade 1–2) in 3 patients, chills (grade 1–3) in 2 patients, and fever (grade 1) in 2 patients. Overall, the safety profile supports the feasibility of using third-party NK cells as a therapeutic strategy in this patient population (Table 2).

**Table 2:** Adverse events and severity grades observed after Third-Party NK-Cell infusion in High-Risk AML patients

NCI CTCAE term	Total, *n*	Grade 1, *n*	Grade 2, *n*	Grade 3, *n*	Grade 4, *n*	Grade 5, *n*
Chills	2	1	1		**-**	**-**
Nausea	1	1	-	**-**	**-**	**-**
Headache	1	-	1	**-**	**-**	**-**
Vomiting	-	-	-	**-**	**-**	**-**
Fever	2	2	-	**-**	**-**	**-**
Sinus tachycardia	3	2	1	**-**	**-**	**-**
Bone pain	-	-	-	**-**	**-**	**-**
Pain in extremity	-	-	-	**-**	**-**	**-**
Rash maculopapular	-	-	-	**-**	**-**	**-**

### Post-HSCT complications and outcomes

Post-transplant complications included mucositis (grade 2–4) in 7 patients (63.6%), acute GvHD (grade 1–2) in 4 patients (36.4%), and chronic GvHD (limited/extensive) in 4 patients (36.4%). Cytomegalovirus (CMV) reactivation occurred in 5 patients (45.5%), with onset between 5 and 63 days post-HSCT. All patients achieved engraftment, with median neutrophil recovery at 13 days (range: 10–17) and platelet recovery at 11 days (range: 8–15) (Table 3).

**Table 3:** Post-Transplant outcomes and complications by patient

Patient		ANC engraftment day	PLT engraftment day	Mucositis	aGvHD	cGvHD	HC	CMV reactivation	Relapse status	Survival status	Cause of death	TTR (day)	FU (day)	Disease free FU (day)
P-1		17	11	-	-	-	-	-	Relapse	Alive	-	159	479	159
P-2		13	9	Grade2	Grade1	limited-moderate	-	-	No R	Alive	-	-	482	482
P-3		13	12	Grade2	-	-	-	-	Relapse	Dead	Relapse	34	54	34
P-4		13	13	Grade2	-	-	-	Positive	No R	Alive		-	256	256
P-5		12	10	-	Grade 2	-	-	Positive	No R	Dead	PTLD	-	144	144
P-6		11	9	-	-	Advanced- moderate	Grade4	-	No R	Dead	Infection	-	210	210
P-7		12	12	-	-	-	-	Positive	No R	Alive	-	-	514	514
P-8		10	11	Grade4	Grade 2	limited- moderate	Grade1	Positive	No R	Alive	-	-	463	463
P-9		14	13	Grade3	-	-	-	-	No R	Alive	-	-	132	132
P-10		13	8	Grade3	Grade 2	limited- mild	-	Positive	Relapse	Dead	Relapse	111	234	111
P-11		15	15	Grade3	-	-	-	-	No R	Dead	Graft Failure	-	166	166

*ANC* Absolute Neutrophil Count, *aGvHD* Acute Graft versus Host Disease, *cGvHD* Chronic GvHD, *HC* Hemorrhagic cystitis, *CMV* Cytomegalovirus, *FU* Follow-up, *PTLD *Post-transplant lymphoproliferative disorders, *R* Relapse, *TTR* Time to relapse

Relapse occurred in 3 patients (27.3%), with a median time to relapse of 111 days (range: 34–159). At a median follow-up of 234 days (range: 54–514), 6 patients (54.5%) remained alive, while 5 (45.5%) died due to relapse (*n*=2), graft failure (*n*=1), infection (*n*=1), or post-transplant lymphoproliferative disorder (*n*=1) [19].

One patient (P-5) developed post-transplant lymphoproliferative disorder (PTLD), described in detail elsewhere. In summary, he was a 45-year-old man with high-risk AML transplanted while not in remission. Following haploidentical HSCT with Flu/Bu conditioning and CSA/MTX prophylaxis, he developed grade 2 acute GVHD and CMV reactivation, later followed by EBV-positive extra nodal NK/T-cell lymphoma on day + 108. Despite reduced immunosuppression and attempted chemotherapy, the disease progressed, and he died on day + 144. This rare NK/T-cell PTLD highlights the need for careful post-transplant surveillance for secondary lymphoid malignancies [19].

## Discussion

The findings of this Phase 1 trial demonstrate the feasibility and an acceptable safety profile for prophylactic third-party NK cell infusions in high-risk AML patients post-HSCT. While relapse occurred in 36.4% (*n* = 4/11) and 54.5% (*n* = 6/11) survived at a median follow-up of 256 days, the uncontrolled design precludes efficacy conclusions. Nevertheless, these outcomes contextualize historically high relapse rates (40–60%) in similar cohorts [3, 20].

Critically, survival correlated strongly with remission status at HSCT: 83.3% (5/6) of patients in CR1/CR2 survived versus only 20% (1/5) with active disease (*p* = 0.02). This mirrors Bjorklund et al. [21] and underscores that baseline disease burden likely dominates outcomes—future studies must stratify by MRD status.

The safety profile was reassuring: No grade ≥ 3 infusion toxicities occurred, and GVHD rates (36.4% acute GVHD grade 1–2; 27.3% chronic GVHD) align with expected post-HSCT sequelae [20]. CMV reactivation (45.5%) was manageable with preemptive therapy, though NK-viral interactions warrant deeper mechanistic study.

Third-party cells compared to donor-derived NK, our relapse rate (36.4%) parallels Ciurea et al.‘s donor-derived NK study (32%) [13], hinting that third-party cells may offer logistical advantages without sacrificing anti-leukemic potential. Donors selected for the “mismatch” at HLA loci to drive favorable NK cell–mediated effects. However, the impact of KIR mismatches, unanalyzed in this cohort, could refine patient selection [11, 22].

Further context is provided by our group’s prior Phase I trial in refractory/relapsed AML (Ahmadvand et al. [23]), where third-party KIR-matching NK cell infusions demonstrated safety with no grade 2–5 toxicities and achieved stable disease in 66.6% of patients who were not HSCT candidates. Our current approach utilizes third-party NK cells without KIR matching in a distinct post-HSCT prophylactic setting. The 83.3% survival in our CR1/CR2 cohort suggests enhanced clinical potential when NK therapy bridges the immune gap early post-transplant, reinforcing the need for disease-phase-specific strategies.

In parallel with our current efforts in AML, we previously conducted a Phase I study evaluating the safety and feasibility of allogeneic NK cell infusion in high-risk lymphoma patients following autologous HSCT [24]. In that trial, NK cell therapy was well tolerated without IL-2 supplementation, and no cases of GVHD, CRS, or neurotoxicity were observed. Notably, 87.5% of patients achieved complete responses, and one had stable disease, underscoring the potential of NK cells to mediate antitumor effects in chemo-refractory settings. These findings collectively support NK cell immunotherapy as a broadly applicable platform across hematologic malignancies, including both lymphoid and myeloid lineages, and further validate the rationale for optimizing NK cell dose, schedule, and combinatorial strategies in post-transplant relapse prevention.

The optimal timing of NK cell infusion remains an area of active investigation. Two main strategies have emerged: pre-transplant cytoreductive NK infusion and early post-transplant prophylaxis. Pre-transplant NK therapy, as demonstrated by [25] and Kulkarni et al. [26], can reduce leukemic burden and improve disease control in refractory AML before conditioning. Conversely, our approach, administering NK cells early after HSCT, targets the vulnerable phase of immune reconstitution to reinforce graft-versus-leukemia effects and eradicate residual disease. Both strategies may be complementary rather than competitive, with timing tailored to disease status: cytoreductive NK infusions for active or refractory disease, and post-transplant NK prophylaxis for patients in remission but at high relapse risk. Future trials directly comparing or sequentially combining these approaches could clarify the most effective integration of NK therapy within the HSCT timeline.

A key aspect of this study was to evaluate the operational feasibility of this approach. While the consistent ~ 500-fold expansion yield of our feeder cell line necessitated the use of multiple third-party donors to ensure adequate cell product, this strategy proved effective. Furthermore, the treatment protocol was manageable, with all eleven patients who proceeded to infusion successfully receiving both planned NK cell doses without delay. Two enrolled patients did not receive infusions due to intercurrent complications (infection and hepatic veno-occlusive disease), which is consistent with the expected attrition in a high-risk post-HSCT cohort. Overall, these findings confirm the feasibility of manufacturing and administering early prophylactic third-party NK cell infusions in this setting.

Several limitations demand caution: The small cohort (*n* = 11), heterogeneous donor types (45% haploidentical), and lack of a control group limit causal interpretation. Additionally, tracking the persistence of the infused NK cells in vivo was not performed; such data in future studies would provide valuable insight into the relationship between cell longevity and clinical outcomes. The outcomes in CR1/CR2 patients highlight a subgroup for prioritized investigation in future trials. Larger randomized studies should test standardized products (e.g., cytokine-activated [27] or CAR-NK cells [28]) against the standard of care.

Future directions should focus on advancing NK cell therapy through engineering, optimized timing, and refined donor selection. NK cell engineering, particularly CAR-NK therapy, could enhance cytotoxicity against resistant clones while minimizing GVHD risks by targeting leukemia antigens via chimeric receptors; early trials suggest these cells retain innate antitumor functions with superior persistence [28, 29].

Furthermore, optimizing the timing of infusion to align with critical immune reconstitution windows, such as the pre-engraftment phase, may maximize therapeutic impact. Concurrently, the strategic integration of KIR-matching could better leverage “missing-self” recognition biology to augment anti-leukemic activity [22]. Combining these advanced approaches with existing HSCT protocols holds transformative potential for improving outcomes in high-risk AML.

## Conclusion

Prophylactic third-party NK cell infusions following HSCT are possible and well accepted in high-risk AML patients; they have acceptable GVHD and viral reactivation. Survival was significantly associated with remission state at transplant, therefore emphasizing the need for CR1/CR2 patients. Although effectiveness cannot be certainly proven in this small, unrestrained group, results match past data and point to third-party NK cells offering logistical benefits without compromising antilemic activity. Future research should concentrate on maximizing NK cell engineering, timing of administration, and KIR-based donor selection to improve relapse prevention techniques.

## Supplementary Information

Supplementary Material 1.

## Data Availability

The datasets generated during the current study are available from the corresponding author at reasonable request.

## Abbreviations

AML: Acute myeloid leukemia
HSCT: Hematopoietic stem cell transplantation
NK: Natural killer
GvL: graft-versus-leukemia
GvHD: Graft-versus-host disease
OS: Overall survival
DFS: Disease-free survival
MRD: Measurable residual disease
PBMCs: Peripheral blood mononuclear cells
PBS: Phosphate buffer saline
MACS: Magnetic-activated cell sorting
IL-2: Interleukin-2
IL-15: Interleukin-15
GMP: Good Manufacturing Practice
