# Supplementary material for: Evaluating the safety and feasibility of prophylactic third-party NK cell administration in high-risk AML patients post-HSCT
Source: BMC Cancer. 2025 Nov 22;25:1931. doi: 10.1186/s12885-025-15362-8 (PMC12752282; doi:10.1186/s12885-025-15362-8)
Supplement: Supplementary file 1 — Supplementary Material 1. [file 12885_2025_15362_MOESM1_ESM.docx]

**
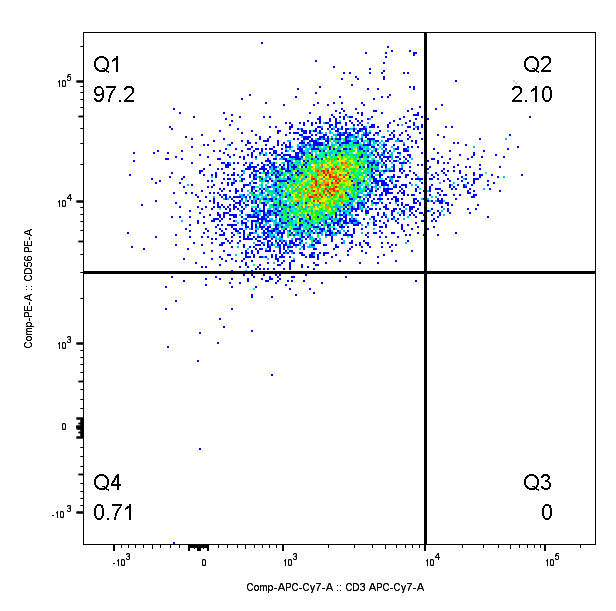
**

**Figure S1.** Expansion and Functional Characterization of PBMC derived third-party NK Cells. (a) flow-cytometry and frequency of CD56^+^CD3^-^ NK cells derived from PBMCs.


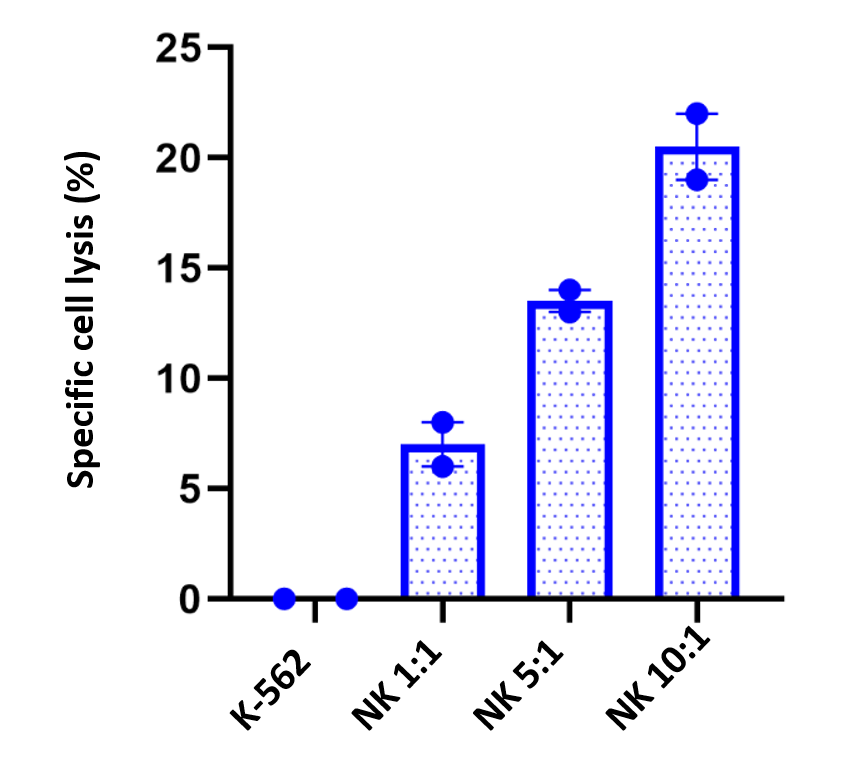


**Figure S2.** Cytotoxicity of activated third-party NK cells (E) against K-562 cells (T) using LDH assay. All data are presented as means ± standard deviation (M±SD, n=3). **P ≤ 0.01, and ***P ≤ 0.001.
